# How we treat mature B-cell neoplasms (indolent B-cell lymphomas)

**DOI:** 10.1186/s13045-020-01018-6

**Published:** 2021-01-06

**Authors:** Melissa Lumish, Lorenzo Falchi, Brandon S. Imber, Michael Scordo, Gottfried von Keudell, Erel Joffe

**Affiliations:** Lymphoma Service, Department of Medicine, Memorial Sloan Kettering Cancer Center, 1275 York Avenue, SR‐441B, New York, NY 10065 USA

**Keywords:** Indolent lymphoma, Mature B cell neoplasm, Follicular lymphoma, Marginal zone lymphoma, Active surveillance

## Abstract

Mature B cell neoplasms, previously indolent non-Hodgkin lymphomas (iNHLs), are a heterogeneous group of malignancies sharing similar disease courses and treatment paradigms. Most patients with iNHL have an excellent prognosis, and in many, treatment can be deferred for years. However, some patients will have an accelerated course and may experience transformation into aggressive lymphomas. In this review, we focus on management concepts shared across iNHLs, as well as histology-specific strategies. We address open questions in the field, including the influence of genomics and molecular pathway alterations on treatment decisions. In addition, we review the management of uncommon clinical entities including nodular lymphocyte-predominant Hodgkin lymphoma, hairy cell leukemia, splenic lymphoma and primary lymphoma of extranodal sites. Finally, we include a perspective on novel targeted therapies, antibodies, antibody–drug conjugates, bispecific T cell engagers and chimeric antigen receptor T cell therapy.

## Introduction

Mature B cell neoplasms, commonly known as indolent non-Hodgkin lymphomas (iNHLs), are a heterogeneous group of malignancies sharing similar disease courses and treatment paradigms. While these lymphomas are generally considered incurable, most patients can expect a lifespan similar to that of the age-matched population, with the exception of those who are young at diagnosis and those who, following initial systemic treatment, progress rapidly or experience transformation into aggressive lymphomas (8–10% risk at 5 years) [1–9]. A major premise in managing iNHL is that earlier treatment does not improve survival, and many patients can be observed for years before treatment is indicated [10–12].

Providing an exhaustive review of each iNHL is beyond the scope of this paper, and excellent disease-specific reviews have been previously published [13–19]. In this review, we will focus on general concepts in the treatment of iNHLs, with specific attention to unique histologies and scenarios in which management should depart from common paradigms.

## Localized disease

As lymphocytes populate nearly every organ system, the occurrence of an isolated nodal or extranodal site of disease is intriguing. It raises the question of whether localized presentation is a matter of chance (i.e., incidental early identification of a process bound to disseminate with time) or represents an inherent biological property “directing” lymphoma to a distinct region. The latter hypothesis is supported by similarly low rates of progression in rigorously staged patients with localized follicular lymphoma (FL) treated with observation compared with radiotherapy (RT) [20]. Further, several studies have suggested that localized FL is often characterized by a unique genetic profile, lower rates of BCL2/IGH translocation and a higher dependence on local microenvironmental features [21–24]. Similar observations apply to unique iNHL histologies involving extranodal sites (see Unique anatomical presentations below) [25–27].

### Radiotherapy

Localized disease, present in 15–30% of patients, is one of the few situations in which iNHL is considered curable. Following RT, over 90% of patients will achieve a complete response (CR) and very few recurrences occur within the irradiated field [28, 29]. Approximately 50% will remain free from progression at 10 years with few relapses occurring beyond that time (i.e., suggesting cure) [30–34]. Current-day response rates may be considerably higher due to improved staging with positron emission tomography (PET), molecular testing of bone marrow (BM) and modern improvements in RT delivery with reduced toxicity [31]. Nonetheless, fewer than 30% of eligible patients are treated with RT despite its curative potential, in part due to skepticism about RT safety [35–37]. Contemporary treatment paradigms utilize involved-site radiotherapy (ISRT), which focuses treatment on radiographically apparent areas of disease. Compared to historical paradigms of total lymphoid irradiation or involved-field RT (IFRT), ISRT has significantly reduced the exposed anatomic areas [38, 39]. Depending on the irradiated site, less than 3% of patients will develop severe acute toxicities and less than 1.5% will develop late toxicities. The most common side effects are local skin erythema and mucositis, which are usually manageable with supportive care strategies and resolve within a few weeks (Table 1). Concerns about secondary malignancies in irradiated patients have not been supported in the population of patients with FL and marginal zone lymphoma (MZL) treated with RT [40–43]. Another possible explanation for the low utilization rate of RT is a sentiment among some clinicians that active intervention is not justified considering the excellent outcomes seen with observation (with up to 20% experience spontaneous remission) [44]. However, a SEER analysis of 6568 patients with localized grade 1–2 FL found that upfront RT was associated with improved disease-specific survival (DSS) and overall survival (OS) compared to patients who did not receive RT (10 y and 20 y DSS 79% and 63% for RT vs. 66% and 51% for no RT) [45]. In contrast, several reports suggested similar OS for upfront RT compared with observation; most notably, a multicenter retrospective review of 256 patients with FL rigorously staged with PET and a BM biopsy and treated with RT (*n* = 171) or watchful waiting (WW) (*n* = 85) demonstrated no difference in the time to first chemotherapy (TTC) treatment (4-year TTC 75–80%) [20, 37, 46, 47].

**Table 1:** Reported radiation-associated toxicities from two randomized, phase 3 trials

Study	Study population	Acute toxicities			Late toxicities		
		Toxicity	All grades (%)	Moderate–severe grade (%)	Toxicity	All grades (%)	Moderate–severe grade (%)
BNLI Dose Reduction [29] (2011)	179 patients with indolent NHL receiving 24 Gy as part of randomized, phase III dose reduction study	Erythema	34	8	Xerostomia	23	8
		Mucositis	25	11	Skin fibrosis	17	2
		Dry desquamation	13	1	Alopecia	16	3
		Nausea/ vomiting	11	4	Mucosal injury	9	4
		Diarrhea	9	1	Cutaneous telangiectasia	8	0

		Toxicity		Grade 3 (%)	Toxicity		Grade 3 (%)
FORT Study [125] (2014)	299 patients with MZL or FL receiving 24 Gy as part of randomized phase III non-inferiority study of very low dose RT	Any		2.8	Any		1.4
		Mucositis		0.7	Mucosal injury		0.7
		Fatigue		0.7	Fatigue		0.4
		Pain in irradiated area		0.4	Pressure sore		0.4
		Diarrhea		0.4	Constipation		0.4

This table outlines the toxicities associated with radiation therapy based on two randomized, phase 3 trials. Note that the FORT study only reported severe toxicities and likely used more modern technology, while the BNLI study reported toxicities of all grades and is based upon data using older techniques

*FL* follicular lymphoma, *MZL* marginal zone lymphoma, *NHL* non-Hodgkin lymphoma, *RT* radiotherapy

A more challenging question is whether patients with a localized major site of disease and minimal systemic involvement (for example, minute BM involvement) benefit from RT, which would no longer offer curative intent. Overall, approximately 40–70% of patients with FL, 4–10% of those with mucosa-associated lymphoid tissue (MALT) lymphoma, 30–60% of those with other MZL, and 50–90% of those with mantle cell lymphoma (MCL) will have morphologic BM involvement [48–57]. Molecular involvement of BM by flow cytometry or PCR, regardless of the presence or absence of morphologic involvement, can be seen in 40–65% of patients with FL at diagnosis, including in some with stage I/II disease. Overall, these patients have a much shorter PFS after RT (~ 5-year PFS of 50% vs. 95%; *n* = 67) and after systemic chemoimmunotherapy (CIT) (3-year PFS 40% vs. 85%; *n* = 53), comparable to that of patients with morphological BM involvement; however this difference is not significant among patients with limited stage disease [58–60]. Similarly, some patients with FL will have evidence of minimal residual disease (MRD), defined as detectable circulating BCL2/IGH + cells, at diagnosis despite negative molecular BM assessment [58]. Although half of these patients will become MRD-negative after RT, this is not associated with a decreased chance of relapse, and MRD-driven consolidation with rituximab after IFRT can improve PFS for these patients [58].

Overall, we favor ISRT for localized disease based on its low toxicity and probable PFS benefit. In the modern era, we treat most localized cases with definitive intent with 24 Gy in 12 fractions based on the noninferior outcomes from the BNLI RT dose de-escalation study [29].

Outcomes of patients who progress after primary RT are favorable, with a 3-year freedom from progression (FFP) of 57% [61]. In certain cases  in which RT toxicity may be higher or a long course of RT cumbersome (e.g., orbital MALT, salivary gland MALT), we offer patients the option of a stepwise approach starting with very low dose RT regimen of 2 Gy × 2 based on promising data from a single institutional series (see RT for symptomatic advanced-stage disease) [62, 63]. Though several authors have advocated a considerable PFS advantage for CIT with or without RT over RT alone, particularly for localized bulky disease (defined in this context as > 5 cm), this is not our practice [64, 65]. At our institution, we rigorously stage patients with PET, incorporating this imaging modality into our RT simulation scan. We treat proximal stage II disease in a single field using modern RT approaches (i.e., intensity modulated RT, IMRT). If a patient does have more distant stage II disease, we will often treat two or more sites using separate isocenters. Given our recommended doses of typically  less than 30 Gy, we rarely encounter dose-limiting toxicity. Although some will be considered for consolidative systemic therapies after RT, many patients will be cured with RT alone. Similarly, we rarely add RT or CIT consolidation for fully resected disease [47]. We do not routinely offer proton RT, which theoretically has improved conformality with lower RT exposure to adjacent tissue, as efficacy data are limited, there can be some uncertainty of dose range near critical normal structures and toxicity of ISRT using IMRT is not a major concern [66].

## Systemic disease

### Indications for treatment

Multiple studies have demonstrated that early treatment of asymptomatic patients with low-burden iNHL does not prolong OS or reduce the rate of histologic transformation (HT) compared to expectant management [11, 12, 67, 68]. Therefore, most clinicians observe patients until conventionally defined treatment criteria are met. Typically, treatment is indicated for patients with symptomatic or high-burden disease and those with end-organ compromise, based on early studies in FL by the Groupe d’Etude des Lymphomes Folliculaires (GELF) and British National Lymphoma Investigation (BNLI). Symptomatic/high burden nodal disease is defined as any mass > 7 cm; involvement of ≥ 3 nodal sites each ≥ 3 cm; splenomegaly below the umbilical line; pleural or peritoneal effusion; cytopenias; and/or > 5.0 k/mcl circulating blood lymphoma cells [11]. Some also consider macroscopic involvement of cortical bone, kidneys and liver, and rapid progression over the preceding 3 months to be indicators of more aggressive disease, justifying the initiation of therapy [12, 69]. While these criteria are based on data from the pre-rituximab era, they seem to hold true in current-day practice [11, 12, 70]. They have also been extrapolated to the management of patients with low-risk MCL, nodular lymphocyte-predominant Hodgkin lymphoma (NLPHL), Waldenstrom macroglobulinemia (WM) and MZL with the exception of presence of circulating lymphoma cells in the peripheral blood in the leukemic iNHLs [71–74]. Reassuringly, none of the aforementioned studies demonstrated a significant increase in the risk of HT in patients initially observed compared to those who were treated early.

Lack of survival advantage, however, does not necessarily imply lack of benefit. In defined groups of patients, early anti-CD20 monotherapy can be relevant. In some elderly or frail patients with progressive, low-burden disease, early treatment with single-agent rituximab may prolong the chemotherapy-free interval. Rituximab can also have a role in the treatment of patients whose quality of life (QoL) is heavily affected by an expectant approach and/or for whom the lymphadenopathy is cosmetically burdensome [75]. In these patients, rituximab monotherapy is associated with a 71% overall response rate (ORR), 12% complete response (CR) rate and median time to treatment failure (TTF) of 4 years, which may be prolonged by adding maintenance therapy or retreating at progression [76].

Concerns associated with the early use of rituximab include the slightly increased risk of infection (1% for rituximab induction vs. 4% for rituximab maintenance) and future resistance to anti-CD20 agents [75]. In the RESORT trial, which evaluated frontline treatment with rituximab monotherapy followed by rituximab maintenance (RM), the additional exposure to rituximab  was not associated with later resistance; however, this was not a research question addressed by the trial [76, 77]. Further data may be provided by a phase III trial comparing WW with rituximab as first-line treatment in patients with advanced-stage FL with low tumor burden (FLORA study, JCOG1411) [78].

In our practice, we do not recommend treatment initiation for asymptomatic patients with advanced, low-burden iNHL because of the lack of survival benefit, because 20–30% of patients will not meet treatment criteria for at least 10 years, and the median time to treatment in newly diagnosed patients is 3 years [12]. Although some clinicians believe that early treatment may instill a sense of security in patients, thus improving QoL, in our experience, patients’ QoL is generally preserved with careful, regular follow-up, judicious use of imaging, transparent communication and proactive management of anxiety and/or depression. In the rare instances in which we consider early treatment with rituximab monotherapy for FL, we generally administer 4 weekly doses without RM, though may consider a second round of rituximab at weeks 15–18, particularly in patients with a partial response (PR) [79]. In those with MZL, LPL/WM and other rare iNHL, such as NLPHL, we adopt a similar approach, but consider RM following induction [71–74, 76, 77].

### Risk prediction and prognostic genomic biomarkers

Several studies have sought to identify clinical and molecular markers of worse prognosis in iNHL, mostly borrowing from FL. The original Follicular Lymphoma International Prognostic Index (FLIPI) was developed based on a cohort of 4167 patients followed in the pre-rituximab era and subsequently validated with current treatment approaches [80–82]. It is based on measures of disease burden (stage, > 4 LN regions), measures of disease activity or end-organ damage (hemoglobin < 12, LDH > upper limit of normal), and patient factors (age > 60). FLIPI2, an updated version developed in the rituximab era, uses BM involvement and largest mass > 6 cm in lieu of stage and nodal regions, and beta-2-microglobulin (β2M) instead of LDH [60]. Both studies used these factors to generate three risk groups: low risk (0–1 risk factors), intermediate risk (2 risk factors), and high risk (3–5 risk factors) with respective 5 y OS by FLIPI of 91% versus 78% versus 51%, and 10 y OS of 71% versus 51% versus 36% [80]. The PRIMA-PI uses only two risk factors (β2M > 3 mg/L and BM involvement) to derive similar risk groups [82]. Poor performance status, which is not part of FLIPI, FLIPI2, or PRIMA-PI, should be considered a negative prognostic feature and was not included due to few patients represented in the cohorts [83].

Finally, m7-FLIPI adds mutational data of seven key genes, four with strong prognostic power (EZH2 and ARID1A—‘good’; EP300, FOXO1—‘poor’) and three that are borderline (MEF2B—‘good’; CREBBP, CARD11—‘poor’) [83]. The m7-FLIPI applies only to patients treated with R-CHOP (rituximab, cyclophosphamide, doxorubicin, vincristine, prednisone) or R-CVP (rituximab, cyclophosphamide, vincristine, prednisone), refining the identification of high-risk patients by reallocating about a third of high-risk per FLIPI alone to the low-risk group. Per m7-FLIPI, patients with high-risk FLIPI are high risk only if there is a ‘poor’ mutation or poor performance status, while patients with EZH2 mutations (~ 20–25% of FLs) are nearly uniformly assigned low risk [83]. The strength of this score is in its negative predictive value, whereby 90% and 80% of low-risk patients can expect to be progression free at 1 and 2 years, respectively, while about 50% of high-risk patients will be refractory to treatment or experience progression of disease (POD) during that time. Importantly, the inclusion of mutations that may be associated with specific clinical features (high turnover and elevated LDH) in the prognostic score may underestimate the prognostic effect of the underlying genomic features. Further, the m7-FLIPI was developed on a small cohort and its prognostic value may not be reproducible in patients treated with diverse regimens (e.g., EZH2 mutations seem to confer better prognosis with R-CHOP but not bendamustine rituximab—BR) [84, 85].

Prognostic scores in other iNHL are not as stringently validated but tend to follow the same logic of incorporating measures of disease burden, disease activity and patient age and performance status. For splenic MZL (SMZL), scores include hemoglobin, platelets, LDH and presence of extra-hilar lymphadenopathy; for MALT lymphoma, age, stage and LDH; and for WM, scores also incorporate IgM levels and β2M [86–89]. In practice, these risk scores cannot be used to guide earlier treatment and are not accurate enough to provide patient counseling [90]. We use these scores to ensure a diligent workup of high-risk patients to rule out an underlying aggressive lymphoma.

### Frontline treatment

In patients meeting criteria for treatment, regimens are based on a backbone of an anti-CD20 agent (rituximab, R; ofatumumab; obinutuzumab, G). In the case of FL, anti-CD20 therapy is combined with either chemotherapy (BR/G or R/G-CHOP) or R-lenalidomide. Regarding the initial choice of anti-CD20 agent, the GALLIUM study compared R-chemotherapy to G-chemotherapy in 1202 patients with FL and demonstrated longer PFS with obinutuzumab (3 y PFS 80% vs. 73%), though this advantage was driven by the bendamustine subgroup and by patients younger than age 60 [91, 92].

As for choice of chemotherapy backbone, bendamustine-based treatment resulted in a 5-year PFS of 65%, compared with 56% for CHOP-based therapy (Table 2) [93–96]. Initial findings from the STiL trial suggested superiority of BR over R-CHOP, particularly in MCL, FL, and WM, but not in MZL [96]. However, these findings were not reproduced in the larger GALLIUM trial, and STiL has been criticized for the crossover design potentially leading to underestimation of the activity of R-CHOP [93]. Nonetheless, it is clear that bendamustine-based treatment is not inferior to CHOP [91, 95]. One concern with bendamustine is the increased though statistically insignificant risk of nonrelapse mortality demonstrated in the long-term follow-up of the BRIGHT and GALLIUM studies (5% vs. 2% GALLIUM and 11% vs. 7% BRIGHT) [91, 95]. Importantly, this statistically insignificant risk was not driven by infection and a significant proportion of deaths in the bendamustine groups were noted several years from treatment. Thus, it is hard to interpret these observations, particularly in the setting of lower rates of progression with bendamustine-based treatment leading to no difference in overall survival. One scenario in which oncologists often favor R/G-CHOP is in suspected yet unproven HT, in those with high LDH or high FDG avidity on diagnostic PET. However, in a recent report from the GALLIUM study, there was no association between treatment arm and subsequent transformation in patients with high standardized uptake value (SUV) at baseline (assessed for SUVmax > 10 and for SUVmax > 20) [97].

**Table 2:** Frontline treatment for localized and advanced-stage indolent lymphoma

Drug	Histology	*N*	Follow-up (y)	Responseˆ^†^: PFS^**^, OS, ORR, CRR	Toxicities^†^ and comments
Localized					
RT (40–45 Gy) versus low-dose RT (24 Gy) [29] (BNLI)	FL, MZL	361	6	RT: 5yOS 73%, ORR 93%, CRR 79% Low-dose RT: 5yOS 74%, ORR 92%, CRR 82% PFS: NS, OS: NS	Low-dose RT: trend for reduced toxicity (Table 1)
RT (24 Gy) versus VLDRT (4 Gy) [125] (FORT)^‡‡^	FL, MZL	548	2	RT: ORR 91%, CRR 68% VLDRT: ORR 81%, CRR 49%	RT: G3–4 3% VLDRT: G3–4 1% (Table 1)
IFRT ± RCVP [64]	FL	150	10	IFRT: 10yPFS 41%; 10yOS 87% (NS) IFRT + RCVP: 10yPFS 59%; 10yOS 95% (NS)	IFRT: G3–4 2%; RCVP: G3–4 51% HT: 19% overall (NS) Staging: CT and BM
R + IFRT [240] Rx8 + RT(30–40 Gy)	FL	85	5.5	5yPFS 78%; 5yOS 96%	G3–4 AEs: 5% 14/17 relapses outside RT field
R versus CIT ± RT versus RT versus WW [37]	FL	206	5	RT: PFS 72 m WW/R/CIT/CIT + RT: PFS NR	Not reported Staging: CT and BM ± PET
RT versus RT + CIT versus CIT (or R alone) versus WW [20]	FL	365	4	RT: 5yPFS 68%, 5yOS 93% RT + CIT: 5yPFS 72%, 5yOS 95% CIT (or R alone): 5yPFS 86%, 5yOS 91% WW: similar OS to other groups, lower PFS	In-field relapse: RT 1.7%, CIT 5.0%, CIT + RM 0% Distant relapse: RT 20.4%, CIT 10.0%, CIT + RM 4.1% Staging: PET and BM
Early stage (not meeting GELF criteria)					
R + RM versus RR [76] (RESORT)	FL	289	4.5	RR: 3yPFS 50%; TTF 4y (NS); 3y TTNT 84% MR: 3yPFS 78%, TTF 4y (NS); 3y TTNT 95% 5yOS 94% both groups	RR: HT 8 patients MR: HT 6 patients; 1 death PML
Advanced stage (meeting GELF criteria)					
BR versus R-CHOP [96] (StiL)	FL, MCL, MZL, LPL, SLL, Other	514	4	BR: PFS 70 m; OS NS R-CHOP: PFS 31 m; OS NS *MZL: PFS difference NS	BR: lower alopecia (0% vs. 100%), hematologic (30% vs. 68%), infection (37% vs. 50%), neuropathy (7% vs. 29%), stomatitis (6% vs. 19%); higher skin tox (16% vs. 9%)
BR versus R-CHOP/R-CVP [95, 241] (BRIGHT)	FL, MCL, MZL, LPL	447	5	BR: 5yPFS 66%, OS NS, ORR 97%, CRR 31% R-CHOP: 5yPFS 56%, OS NS, ORR 91%, CRR 25%	As above (StiL)
R^2^ versus R-chemo (CHOP/B/CVP) [104] (RELEVANCE)	FL	1030	3	R^2^: 3yPFS 77%, ORR 86% (NS); CR 48% R-chemo: 3yPFS 78%, ORR 92% (NS), CRR 53% OS: NS	R^2^: G3–4 cutaneous (7% vs. 1%) R-chemo: G3–4 neutropenia (32 vs. 50%), febrile neutropenia (2 vs. 7%) 20% of patients not evaluable, PFS may be underestimated
G-chemo (CHOP/CVP/B) versus R-chemo [91] (GALLIUM)	FL	1202	3	G-chemo: 3yPFS 80% R-chemo: 3yPFS 73% OS: NS	G-chemo: G3–5 74.6%, SAE 46.1% R-chemo: G3–5 67.8%, SAE 39.9% PFS driven by younger patients treated with BG versus BR
R ± chlorambucil versus chlorambucil [109]	Extra-nodal MZL (MALT)	401	7	R-chlor: 5yPFS 72%, 5yEFS 68%, ORR 94.7% R: 5y PFS57%, 5yEFS 50%, ORR 78% Chlor: 5yPFS 59%, 5yEFS 51%, ORR 86% OS: NS	HT: 10 patients total (2 chlor, 6 R-chlor, 2 R alone) AEs: no unexpected differences
R ± R maintenance (RM) [107]	NLPHL	39	10 (R), 5 (RM)	R: 5yPFS^‡^ 39.1%, 5yOS^‡^ 95.7% R + RM: 5yPFS^‡^ 58.9% (NS), 5yOS^‡^ 85.7%	HT: 9 of 23 pts with relapse (39%)
Ibrutinib-R versus placebo-R [111] (iNNOVATE)	WM (1st line and relapse)	150	2	Ibrutinib-R: 30mPFS 82%, 30mOS 94% (NS), ORR 92%, CR/VGPR/PR 72%	Ibrutinib-R AEs: diarrhea, arthralgia, nausea; bleeding (51% vs. 21%) Ibrutinib-R G3–4 AEs: HTN (13% vs. 4%), afib (12% vs. 1%)

Review of the landmark studies that guide upfront management of indolent lymphoma, with attention to the sample size studied, median follow-up time, survival and response rates, and toxicities/adverse events for each regimen

*AEs* adverse events, *afib* atrial fibrillation, *B* bendamustine, *BM* bone marrow biopsy, *BR* bendamustine, rituximab, *CIT* chemoimmunotherapy, *CRR* complete response rate, *EFS *event-free survival, *FL* follicular lymphoma, *G* obinutuzumab, *G3–4/5* grade 3–4/5 toxicities or adverse events, *HT* histologic transformation, *HTN* hypertension, *IFRT* involved-field radiation therapy, *LPL* lymphoplasmacytic lymphoma, *m* months, *MCL* mantle cell lymphoma, *MRD* minimal residual disease-negative (blood or bone marrow as noted), *MRR* major response rate, *MZL* marginal zone lymphoma, *NR* not reached, *NS* no significant difference between groups, *ORR* overall response rate, *OS* overall survival, PFS** progression-free survival, *POD24* progression of disease within 24 months of diagnosis, *R-CHOP* rituximab, cyclophosphamide, doxorubicin, vincristine, prednisone, *R-CVP* rituximab, cyclophosphamide, vincristine, prednisone, R^2^ rituximab, lenalidomide, *R* rituximab, *RM* rituximab maintenance, *RR* retreatment rituximab, *RT* radiotherapy, modality not specified, *SAE* serious adverse events (fatal or life-threatening events that cause or prolong in-patient hospitalization or substantial disability), *SLL* small lymphocytic lymphoma, *TTF* time to treatment failure, *TTNT* time to next treatment, *VLDRT* very low dose RT, *WW* watchful waiting, *y* year

^**^PFS that gives time in months or years = median PFS

ˆStatistically significant difference between groups unless reported as NS

^**†**^If not listed, then the outcome was not reported in the original study

^‡^Estimated

^‡‡^25% had received previous RT and 34% received prior chemotherapy in the FORT study

The addition of maintenance anti-CD20 once every 2 months for 2 years is associated with a considerable increase in PFS after frontline and second-line treatment with CHOP-based therapy. The PRIMA study, which evaluated RM in 1018 patients who attained an initial PR or better with R-chemotherapy (mostly R-CHOP), demonstrated that RM was associated with a 6.5-year increase in PFS (median PFS 10.5 vs. 4.1y; 10y PFS 51% vs. 35%) [94]. This benefit was independent of the depth of response (CR vs. PR). However, even without RM, the median time to next chemotherapy was over 9 years, and there was no difference in OS (10y OS ~ 80% in both arms). There was also no difference in the rate of transformation between the arms (8–9%), and although the rate of early progression (24 m from starting chemotherapy) was lower in the RM arm (~ 12% vs. 25%), the limited efficacy of RM in more aggressive disease explains the equivalent OS [98]. Serious infectious complications or lack of response to subsequent treatments after prolonged rituximab exposure was not of significant concern after initial R-CHOP [94]. However, the addition of maintenance after bendamustine-based regimens is controversial and may be associated with an increased risk of severe, sometimes fatal, late infections, particularly in older patients (12–17% vs. 4–6%, fatal in 4–6% vs. 2%) [91, 99]. The role of RM in non-FL histologies is poorly defined with nonrandomized observational studies suggesting a potential benefit in WM and MZL (WM median PFS 56 m with RM vs. 29 m without), however, with increased rates of infection (43% vs. 25%) [100–103].

In FL, lenalidomide has been used in lieu of chemotherapy. The RELEVANCE trial compared R-lenalidomide (R^2^) to R-chemotherapy (72% R-CHOP) in 1030 patients with FL (50% high-risk FLIPI; 13% grade 3A; 40% bulky > 7 cm disease). It demonstrated similar response rates and PFS between regimens (R^2^: ORR 86%, CR 48%, 3y PFS of 77%; R-chemo: ORR 92%, CR 53%, 3y PFS 78%) which may be an underestimation as 20% of patients were not evaluable [104]. Preliminary results from a phase II study suggest outcomes are similar when using obinutuzumab in place of rituximab (*n* = 90; CR by PET 92%; 12% discontinuation rate) [105]. The main drawback of lenalidomide-based treatment is its length of 18–24 months and the prevalent yet manageable low-grade rash, diarrhea and constipation (each in about a third of the patients—for a review of management of these side-effects see [106]. Compared to R-CHOP, there is no hair loss and there are lower rates of febrile neutropenia (2% vs. 7%) [104].

For iNHL other than FL (e.g., MZL, MALT, NLPHL), initial treatment with rituximab monotherapy results in high response rates and long PFS (ORR 73–78%, CR by CT 42–55%, PFS 5–6 years), and incorporation of chemotherapy may be reserved for patients with bulkier disease or in whom a rapid response is warranted [107–109]. The role of lenalidomide in MZL is not clear with a subanalysis from the AUGMENT trial in 63 relapsed/refractory (R/R) cases showing no advantage over rituximab monotherapy (though limited by sample size and imbalance in baseline prognostic factors) [110]. For other lymphomas, including WM or SLL, initial treatment with ibrutinib or venetoclax (in SLL) may be preferred, at a cost of indefinite treatment as opposed to short-term chemotherapy [111, 112].

Finally, patients with hairy cell leukemia (HCL) attain exceptionally good responses with minimal toxicity using a combination of rituximab and cladribine with near-uniform CR and long-term (7–10 years) PFS and MRD negativity can be achieved with minimal toxicities using a combination of cladribine with concurrent rituximab, which is our practice [113, 114]. Durable remissions can also be attained with cladribine or pentostatin monotherapy (CR rate > 75%, PR rate > 5%, 4-year OS > 95%) which is the standard of care in many centers [115–118]. Of note, treatment with purine analogs in this setting may be associated with early profound cytopenias and blood counts should be evaluated regularly during treatment. The role of the BRAF-inhibitor vemurafenib is being explored in the upfront setting for HCL and has shown promising response rates [119].

In summary, frontline R/G-CHOP, B-R/G and R^2^ have comparable response rates in FL and treatment selection should be based on patient-specific factors. We prefer frontline B-R/G for its favorable toxicity profile and short treatment duration but avoid adding maintenance in this setting. In patients who are chemotherapy averse or in whom side effects of cytotoxic chemotherapy would be problematic, we offer R^2^. We consider obinutuzumab in place of rituximab for younger patients with FL (age < 70) particularly in the context of a bendamustine-based treatment [92]. Though still in its infancy, genomic biomarkers may contribute to management decisions. In FL, a recent report from the GALLIUM study suggested superiority of R-CHOP over BR in EZH2-mutated cases (20–25% of the population; HR 0.25) [84, 120]. For patients with *TP53, NOTCH1* and *SF3B1* mutations, targeted agents or clinical trials should be considered [121, 122].

### RT for symptomatic advanced-stage disease

In symptomatic advanced-stage disease, the role of RT is primarily palliative. We rely on a program utilizing very low doses of RT (VLDRT), typically 2 Gy × 2, as compared to the standard full-dose regimens of 24–30 Gy [17, 123]. VLDRT has proven highly effective in controlling lymphomatous lesions irrespective of overall disease stage, histology or number of prior lines of systemic therapy [124]. VLDRT is associated with an ORR of > 80% with an anticipated 5-year local PFS of ~ 75% (though inferior to 24 Gy ORR > 90% and 5yPFS 91%) [125, 126].

We reserve VLDRT, rather than ISRT, for patients who require RT to multiple disease sites, have detectable BM involvement, and/or are frail. We reassess patients 10 weeks after VLDRT and consider additional 4 Gy or escalation to full dose RT depending on the residual disease.

### Imaging and biomarkers for diagnosis and response assessment

Although PET is known to be sensitive and specific for detection of residual disease in aggressive lymphomas, the use of PET in iNHL is controversial due to high variability in FDG-uptake [127, 128]. Baseline PET is important in identifying cases of transformation [129]. Following CIT, response assessment by PET is superior to that by CT, as it identifies more than twice as many patients with a CR (Deauville ≤ 3 consistent with CR in 76% of patients). This is associated with a 30mPFS of 87% compared to 55% for patients with Deauville > 3 [130, 131].

More recently, circulating tumor DNA (ctDNA) has gained attention as a new method of MRD assessment [132]. The advent of high-throughput sequencing (HTS)-based approaches has made it possible to detect small amounts of ctDNA that is continually shed into the bloodstream [133]. Preliminary data in DLBCL, HL and FL suggest that ctDNA has promise as a marker of MRD and predictor of recurrence [134–137]. In an ongoing study, we aim to prospectively analyze ctDNA levels in patients with newly diagnosed FL to benchmark levels in correlation to treatment response with the goal of utilizing this as an early endpoint in future clinical trials (NCT04468841).

### Unique anatomical presentations

While the majority of iNHLs present with predominantly nodal and BM involvement, there are several unique clinical presentations of organ-specific extranodal lymphomas that warrant distinct treatment strategies.

#### Splenic lymphoma and splenectomy

One unique clinical presentation is that of iNHL with circulating peripheral blood lymphoma cells with or without splenomegaly, but with minimal to no lymphadenopathy (LAD). This presentation is characteristic of SMZL, but can also be seen in a subset of chronic lymphocytic leukemia/small lymphocytic lymphoma (CLL/SLL), MCL, HCL and WM [138, 139]. All tend to be characterized by an indolent course and may be associated with shared molecular pathway abnormalities [140]. Treatment paradigms vary between institutions, but in our experience these patients may be managed expectantly in a similar fashion to those with SMZL. For SMZL, some cases are hepatitis C virus (HCV) associated and regression of the MZL can be achieved with antiviral therapy; for this reason, we test patients with newly diagnosed SMZL for HCV prior to proceeding with treatment [141, 142]. Our preference for frontline treatment is rituximab monotherapy, which is associated with a response rate of 95%, 5y PFS 73% (vs. 58% for splenectomy) and 10y PFS of 60%, outcomes comparable to those of rituximab plus chemotherapy [143–145]. We plan for the possibility of splenectomy at diagnosis and if feasible defer any treatment until pre-splenectomy vaccinations for encapsulated bacteria are completed. In patients who fail rituximab, we use BR (ORR 91%, CR rate 73%, 3y PFS 90%) with close observation and referral for splenectomy in those who fail to achieve a good response or progress early [146]. In the latter case, we aim to attain some control of the size of the spleen (preferably < 20 cm craniocaudal axis) to allow for a laparoscopic procedure [147, 148]. The risk of perioperative mortality seems to be lower than anticipated in the case of iNHL, less than 2% in a cohort of nearly 2000 patients [149]. Post-splenectomy infections are noted in approximately 3%, with overwhelming infections in 1.4% of patients, mostly within the first 3 years following surgery [150, 151]. Postsurgical splenic and portal vein thromboses remain relatively common complications of splenectomy, with an average incidence of 2–3% [152]. An alternative to surgery is low-dose splenic radiation (4–8 Gy), which is not anticipated to result in significant adverse events (AEs), particularly if the adjacent kidney is not included in the radiated field [153, 154]. Data about the feasibility of this approach come from a limited historical case series, and a clinical trial in patients who have failed rituximab is about to be open [155].

#### Gastrointestinal (GI)

The GI tract is the most common site of origin of extranodal lymphomas, and secondary GI involvement can occur in approximately 10% of patients [156]. For gastric MALT lymphoma associated with active *H. pylori* infection, eradication of the bacteria can lead to an estimated 10-year OS 95% and EFS 86% (CR rate 50–90%), while in the remainder, gastric radiation is associated with up to 100% CR rate, and 10-year relapse-free survival 77% [157–159]. Of note, 15–30% of patients present with an 11;18 translocation involving the MALT1 gene on chromosome 18 (API2-MALT1) and have a lower response rate to antibiotics and in some cases resistance to rituximab monotherapy [160–162]. The role of other translocations involving the MALT1 gene (e.g., 14; 18) is less clear, though these entities seem molecularly similar. iNHL involving other sites in the GI tract are mostly MALT lymphomas and FL that in most cases are characterized by an exceedingly indolent course [163–165]. Unique in this regard is the rare entity of immunoproliferative small intestinal disease (IPSID, also known as alpha heavy chain disease or Mediterranean lymphoma) which is a variant of MALT which secretes alpha heavy chains. This subtype typically presents in younger male patients, is associated with Mediterranean decent and often manifests with abdominal pain, chronic diarrhea, malabsorption, and severe weight loss [166].

#### Lungs

Primary malignant lymphomas of the lung are very rare. These are mostly extranodal MZLs of bronchial-associated lymphoid tissue (BALT), though primary FL or MCL of the lungs has been reported anecdotally or by extension from widespread disease [167]. Low frequencies of persistent infection with *Chlamydia pneumoniae, C. trachomatis,* and *C. psittaci* have been identified in pulmonary MALT lymphomas, though BALT has not been directly connected to a single pathogenic agent [168]. Although some oncologists may feel compelled to treat BALT early, we recently demonstrated that BALT lymphoma is an indolent disease that can be managed by local excision or expectantly in most patients, without systemic therapy for many years [169].

#### Central nervous system (CNS)

CNS involvement by iNHL occurs in less than 3% of patients [170]. Although it has been most well described in LPL/WM as Bing-Neel syndrome, it can also occur in SLL, MCL and FL [171, 172]. Treatment, which involves systemic and intrathecal chemotherapy, should be considered carefully due to low response rates and high toxicity [173]. Recent data have shown significant response rates to ibrutinib therapy which has been given at a dose of 420 mg as well as 560 mg with or without rituximab, which is our preference in WM, MCL and SLL [174, 175]. Though further investigation is required, focal RT and whole brain RT (WBRT) have been demonstrated safe and effective in some cases [176].

#### Skin

The two main subtypes of cutaneous iNHL are primary cutaneous follicle center lymphoma (PCFCL) and primary cutaneous marginal zone lymphoma (PCMZL). There are two known subtypes of PCMZL, one which demonstrates diffuse proliferation of neoplastic B cells expressing IgM and CXCR3 (clinically similar to MALT lymphoma) and the other which expresses class-switched immunoglobulins and shows a predominance of T cell infiltration [177]. PCFCL is comprised of large cleaved cells (which may be confused with DLBCL) that are usually localized on the head, scalp or trunk, and are mostly BCL2-negative without t(14;18) translocation [178]. Although the underlying etiology of cutaneous lymphomas is unclear, *Borrelia burgdorferi* infection (Lyme disease) has been significantly associated with primary cutaneous lymphomas in endemic geographic regions, though it remains unclear whether treatment of *B. burgdorferi* infection leads to regression of the lymphoma [179, 180]. Both PCMZL and PCFCL are indolent diseases which can be managed expectantly, with localized RT or with rituximab monotherapy [181].

#### Ocular

Extranodal MZL accounts for greater than 50% of ocular adnexal lymphomas (OAL), which are rare and most often localized [182, 183]. Similar to the potential infectious drivers seen in other MZLs, a subset of OAMZL patients will have an underlying *C. psittaci* infection [184]. Although a phase II study including 27 patients with newly diagnosed or relapsed OAMZL treated with doxycycline showed an ORR of 48% and a 2-year failure-free survival of 66%, this is not standard practice [185]. The majority of cases reported in the literature are treated with either standard-dose (24–30.6 Gy) or high-dose (> 30.6 Gy) IMRT with overall response rate greater than 90% and 5-year local control rates approaching 100% and overall survival greater than 90% [183].

## Relapsed and refractory disease

Once relapse is suspected, it is important to rule out HT to a more aggressive lymphoma particularly if progression occurs within the first 12–24 months after prior therapy (POD12/24). POD12 and POD24 have received much traction in recent years as an indicator of more aggressive disease requiring distinct treatment approaches [186]. However, it is important to note that the poor prognosis of POD12/24 is mainly driven by a subset of patients who experience HT (estimated at 30–40% of those with POD24 treated with R-CHOP and 75% of those with POD24 treated with BR) [9, 187–189]. These rates may be lower with the incorporation of PET in the diagnostic workup of iNHL, which allows for early recognition of HT prior to initiation of frontline therapy. [129, 187] Long-term outcomes of patients who experience early progression with an indolent histology are considerably superior, and it is not clear that they should be managed differently than patients with a later progression [129, 187, 190]. After HT has been excluded, patients may be observed for a period of time if they are asymptomatic without signs of end-organ compromise. If low disease volume or comorbidities are present, retreatment with a course of single agent rituximab can be considered, resulting in a median time to treatment failure of approximately 4 years [76, 79, 191]. Substituting rituximab with obinutuzumab in this setting does not translate into an improvement in PFS [192]. Ultimately, in patients meeting criteria for treatment (as with frontline disease), our preference is to choose a noncross-reacting frontline regimen, typically associated with response rates of 85% for R-CHOP, 82% for BR, and 78% for R^2^ (Table 3) [110, 193]. In patients with multiple relapses, we opt for enrollment on a clinical trial or use one of the targeted therapies discussed below.

**Table 3:** Treatment options at progression of disease for indolent lymphoma

Drug	Histology	*N*	Follow-up (y)	Responseˆ^†^: PFS^**^, OS, ORR, CRR	Toxicities and comments
First relapse					
G versus R [192] (GAUSS)	FL (85%), Non-FL (15%)	175	2.5	G: 2yPFS 46%, ORR 44% (FL); CRR NS R: 2yPFS 50%, ORR 33% (FL); CRR NS	SAEs: 26 patients (15% each arm), majority infusion-related
R^2^ versus R-placebo [110] (AUGMENT)	FL (82%), MZL	358	2.5	R^2^: PFS 3y, ORR 78%, CRR 34% R-placebo: PFS 1y, ORR 53%, CRR 18% *MZL: PFS difference NS OS: data maturing	R^2^ AE: G3–4 neutropenia 50%; infxn 63%, cutaneous 32% R-placebo AE: G3–4 neutropenia 13%, infxn 49%, cutaneous 12%
BR versus G + B [242] (GADOLIN)	FL (80–82%), MZL, SLL, WM	413	2	G + B: PFS NR B: PFS 15 m OS and ORR: NS	G + B: G3–5 AEs 68% B: G3–5 AEs 62% (neutropenia, anemia, thrombocytopenia, infusion reaction)
Multiply relapsed					
BR versus fludarabine + R (FR) [193]	FL, LPL, SLL, MZL, MCL	230	8	BR: PFS 34 m, ORR 82%, CRR 40% FR: PFS 12 m, ORR 51%, CRR 17%	SAEs: 46 total (23 each group); myelosuppression, infection
PI3K3-inhbitors					
Idelalisib [197] (PI3Kδ-inhibitor)	FL (59%), SLL, MZL, LPL	64	NA	PFS 8 m, ORR 47%, DOR 18 m TTR: 1 m	AEs: infection; monitor CMV, pneumocystis jiroveci prophylaxis
Copanlisib [196] (CHRONOS-1)	FL (73%), MZL, SLL, LPL-WM, *DLBCL (1%)*	142	NA	PFS 11 m, OS NR, ORR 59%, CRR 15%	AEs: hyperglycemia (50%), diarrhea (35%), fatigue (30%), hypertension (30%), neutropenia (29%), fever (25%) SAEs: lung infection (13%), hyperglycemia (5%), decreased neutrophil count (4%), fever (3%), and diarrhea (2%), pneumonitis (8%) *25% discontinuation, 37% dose reduction, 74% interruptions
Duvelisib [195] (DYNAMO)	FL (64.3%), SLL, MZL	129	3	PFS 10 m, ORR 47% (most PR, 2 patients CR)	AEs: diarrhea (49%), nausea (30%), neutropenia (29%), fatigue (28%), cough (27%); 19% dose reduction, 66% interruption, 31% discontinued
BTK inhibitors					
Ibrutinib [204]	MZL	63	1.5	PFS 14 m, 18mOS^‡^ 81%, ORR 48% TTR 4.5 m, best response 5 m	62% discontinued treatment Pseudoprogression in 2 patients
Ibrutinib-R versus placebo-R [111]	WM (1st line and relapse)	150	2	Ibrutinib-R: 30mPFS 82%, MRR 72%	Ibrutinib-R G3–5 AEs: afib 12%, HTN 13%; IgM flare 8%, infusion rxn 1% Response independent of MYD88 or CXCR4 genotype
Other novel agents: BCL-2 and EZH2 inhibitors					
Venetoclax [210] (BCL-2 inhibitor)	WM	31	NA	2y PFS 76%, ORR 87%, VGPR 19%, PR 61%, TTR 2 m	AEs: G4 neutropenia (*n* = 5), G3 neutropenia (*n* = 15), G3 anemia (*n* = 4), diarrhea (*n* = 4)
Tazemetostat [213] (EZH2 inhibitor)	FL	99	NA	MT EZH2: PFS 14 m, ORR 77% WT EZH2: PFS 11 m, ORR 34%	AEs: G ≥ 3 17% (all patients), most frequent thrombocytopenia (3%), anemia (2%), asthenia (2%), vomiting (1%), fatigue (1%), no G5 AE
Antibodies, antibody–drug conjugates (ADC) and bispecific T cell engagers (BiTE)					
R-Polatuzumab vedotin [216] (CD79b-directed AB-drug conjugate)	FL	20	NA	PFS 15 m, ORR 70%, CRR 45%	G3–5 AEs (50%): neutropenia (15%), diarrhea (10%); one G5 event
Mosunetuzumab [217] (bispecific Ab targeting CD3, and CD20)	FL	69	NA	ORR 64%, CRR 44%	CRS: 28.4% (FL and aggressive NHL), most G1–2 G3 CRS: 1.4%, most CRS in cycle 1
Magrolimab (anti-CD47 Ab)	FL (35%), MZL (2%), *DLBCL (63%)*	100	1–1.5	ORR 61%, CRR 24% TTR 2 m DOR NR	AEs: infusion reactions (38%), headache (34%), chills (30%), fatigue (30%), anemia (27%), nausea (24%), pyrexia (23%) vomiting (13%), back pain (11%); majority G1/2 except G3 anemia (15%)
Auto/allo HSCT and CAR-T					
HDT/ASCT versus no transplant [221]	FL	162	11	ASCT: 5y PFS 51%, OS 77% No transplant: 5y PFS 19%, OS 59%	*Among patients without cytoreduction failure, PFS not significantly different; second-line PFS is reported for patients with POD24
Allo-SCT (MSD or MUD) versus ASCT [222]	FL (R/R)	440	6	ASCT: 5yPFS 38%, 5yOS 70% MSD: 5yPFS 52%, 5yOS 73% MUD: 5yPFS 43%, 5yOS 49% *After 6 m, MSD/MUD PFS > ASCT PFS	ASCT: 5y NRM 5% MSD: 5y NRM 17% MUD: 5y NRM 33%
Axicabtagene ciloleucel [237] (CD19-CAR T) (ZUMA-5)	FL, MZL	94	11.5 m	PFS 23.5 m, OS NR, 12mOS 94%, ORR 94% (79% CR): FL ORR 95% (80% CR), MZL ORR 86% (71% CR)	Grade ≥ 3 AEs 83%: neutropenia (33%) and anemia (28%); grade ≥ 3 CRS: 11%; grade ≥ 3 neurologic events: 19% Median time to CRS: 4d; Median time neurotoxicity: 7d

This table outlines the standard chemoimmunotherapy regimens and novel agents that have efficacy in relapsed or refractory indolent lymphomas. Several of the novel therapies demonstrate excellent response rates with unique toxicity profiles

*Ab* antibody, *AEs* adverse events, *afib* atrial fibrillation, *allo-SCT* allogeneic stem cell transplant, *ASCT* autologous stem cell transplant, *B* bendamustine, *BM* bone marrow biopsy, *BR* bendamustine, rituximab, *CAR T* chimeric antigen receptor T cell, *CIT* chemoimmunotherapy, *CRR* complete response rate, *DOR* duration of response, *EFS* event-free survival, *FL* follicular lymphoma, *G* obinutuzumab, *G3–4/5* grade 3–4/5 toxicities or adverse events, *HDT* high-dose chemotherapy, *HT* histologic transformation, *HTN* hypertension, *LPL* lymphoplasmacytic lymphoma, *m* months, *MCL* mantle cell lymphoma, *MRR* major response rate, *MSD* matched sibling donor, *MT EZH2* mutant enhancer of zeste homologue 2, *MUD* matched unrelated donor, *MZL* marginal zone lymphoma, *NA* not available, *NR* not reached, *NRM* nonrelapse mortality, *NS* no significant difference between groups, *ORR* overall response rate, *OS* overall survival, PFS** progression-free survival, *POD24* progression of disease within 24 months of diagnosis, *R-CHOP* rituximab, cyclophosphamide, doxorubicin, vincristine, prednisone, *R-CVP* rituximab, cyclophosphamide, vincristine, prednisone, *R*^*2*^ rituximab, lenalidomide, *R* rituximab, *RR* retreatment rituximab, *SAE* serious adverse events (fatal or life-threatening events that cause or prolong in-patient hospitalization or substantial disability), *TTR* time to response, *WT EZH2* wild type enhancer of zeste homologue 2, *WW* watchful waiting, *y* year

^**^PFS that gives time in months or years = median PFS

ˆStatistically significant difference between groups unless reported as NS

^**†**^If not listed, then the outcome was not reported in the original study

^‡^Estimated

### Targeted therapies

#### PI3K3-inhibitors

Many iNHLs depend on the phosphatidylinositol 3-kinase delta (PI3Kδ) pathway, and there are now three PI3K-inhibitors (PI3Ki) approved for the treatment of R/R FL after two prior lines of therapy (idelalisib, copanlisib, and duvelisib—overall *N* ~ 450, ORR of 50–60%, CR 5–15%, median PFS 10–12 m in a heavily pretreated population) [95, 194–196]. Responses to PI3Ki should be seen within the first few months of treatment (median ~ 3 m) and are observed at similar rates among patients who had prior POD24 and among varied subtypes of R/R iNHL [197].

Idelalisib, a PI3Kδ-inhibitor, was the first drug to be studied (*N* = 125) and demonstrated an ORR of 57% (6% CR), median PFS 11 m and OS 20 m. Duvelisib, an oral inhibitor of the PI3K-δ and -γ isoforms, received approval based on an open-label, global phase II trial (DYNAMO) (*N* = 129), which demonstrated ORR 47%, median PFS 10 m [195]. Unfortunately, toxicity with both agents is nearly universal leading to drug discontinuation in approximately 25%, dose reduction in 20–40%, and treatment interruptions or delays in 65–75% of patients [195, 196]. Further, there are black-box warnings for severe transaminitis (~ 15%), diarrhea (~ 15%), pneumonitis (~ 5%) and bowel perforation (~ 0.5%) (idelalisib). Other common grade 3 AEs are neutropenia (27%) and increased risk of pneumocystis jiroveci pneumonia (PJP) and CMV reactivation (both rare), which require PJP prophylaxis and regular CMV RNA monitoring. For diarrhea, there are two peaks, early (easily manageable) and late ~ 6 m (which resembles inflammatory colitis). Colitis and transaminitis appear to occur more frequently in patients with fewer prior lines of therapy [198–200]. Transaminitis tends to occur early within the first 3 months (recommendation to monitor laboratories every 1–2 weeks for the first 6m).

Copanlisib, a PI3K α,δ-inhibitor, was evaluated in a phase II study (CHRONOS-1) (*N* = 142) with ORR 59% (CR 15%), median PFS 11 m. Toxicity was again nearly universal leading to drug discontinuation in 25%, dose reduction in 37%, and treatment interruptions or delays in 74% of patients, though most commonly hyperglycemia (50%) or diarrhea (35%). It does not require PJP or CMV prophylaxis and has no black-box warnings [201].

Of note, preliminary data from ME-401 (a novel PI3Ki) suggest that intermittent treatment for 7 days out of a 28-day cycle may offset the risk of severe AEs while maintaining high response rates [202]. Meanwhile, preliminary data for parsaclisib (highly selective PI3Kδ-inhibitor) demonstrate objective response rates of ~ 70% for relapsed or refractory iNHLs [203]. Umbralisib is another PI3Ki under investigation which confers fewer episodes of autoimmune-like toxicities.

#### BTK-inhibition

Ibrutinib, acalabrutinib and zanabrutinib bind covalently at the cysteine 481 site on the BTK receptor and have activity in SLL, WM, and MZL. All rarely lead to a CR, but most patients have stable disease or better and benefit from a 2-year PFS of 80% in WM and a median PFS of 14 months in MZL [111, 204]. In WM, patients with MYD88 mutations and CXCR4 wild type have improved response to ibrutinib, while those with both MYD88 and CXCR4 mutations have an inferior response to treatment which can be offset with the addition of rituximab and patients with wild-type MYD88 and CXCR4 mutations are unlikely to respond [111]. In MZL, the frequency of MYD88 mutation is less than 5% while ORR to ibrutinib 48%, suggesting that responses are independent of MYD88 mutation status [204, 205]. Notwithstanding, in a recent analysis in R/R MZL, patients with TNFAIP3 mutations had better responses to ibrutinib and those with MYD88L265P mutations had longer PFS [206]. Conversely, patients with mutations in KMT2D and CARD11 derived less benefit from treatment. CARD11 mutations have also been demonstrated in association with primary resistance to ibrutinib in several other studies, while development of mutations at the C481 site of BTK and in PLC-gamma is associated with acquired resistance to this class of drugs [207, 208]. To overcome these mechanisms, there are novel agents currently in clinical trials which target BTK in a noncovalent manner, have additional cellular targets (e.g., SRC kinases) or have targets downstream from MYD88 such as IRAK-4 and MALT1 (NCT03740529, NCT03893682, NCT03328078, NCT03900598, NCT03162536).

#### BCL-2 inhibition

BCL-2 is an anti-apoptotic protein implicated in the pathobiology of many iNHLs, most known in the context of the BCL2:IGH (t14;18) translocation in FL. Venetoclax, an oral BCL2 inhibitor, has considerable activity in CLL and MCL, but surprisingly disappointing activity in FL (ORR 38%, CR 14% PFS 13 m) [209]. Evaluation in other iNHL has been limited, though the drug is expected to have activity in MZL and WM [210]. Importantly, the drug has a manageable toxicity profile [209, 211]. To promote activity in FL, several trials are evaluating combination therapy with PI3K inhibition and with epigenetic targeting with EZH2 inhibitors.

#### EZH2 inhibition (FL)

Next-generation deep sequencing has uncovered mutations in chromatin-modifying genes (CMG), including KMT2D, CREBBP, and EZH2 in the majority of FL patients, which provide attractive therapeutic targets [212]. Tazemetostat, a drug targeting EZH2, has become one of the most promising novel therapeutics in FL with an ORR 77% in *EZH2mut* and 34% in *EZH2wt*, median PFS of 14 months and 11 months, respectively, and a manageable toxicity profile [213]. The drug has been recently approved by the FDA in patients with relapsed FL who have failed at least two lines of therapy. Several ongoing trials are evaluating EZH2i in combination with other targeted agents (NCT04224493, NCT02601950).

### Antibodies, antibody–drug conjugates (ADC) and bispecific T cell engagers (BiTE)

Several novel antibodies, ADCs and BiTEs are in advanced stages of clinical trials and have been recently recommended by the NCCN guidelines. These include the anti-CD79b ADC polatuzumab vedotin, the CD3-CD20 BiTE mosunetuzumab and the anti-CD47 antibody magrolimab (Hu5F9-G4). Polatuzumab is NCCN recommended in FL and MCL (but not MZL) for use after ≥ 2 prior lines of therapy with rituximab (ORR 70%, CR 45%, median PFS 15 m; *N* = 20) or in combination with BR (CR by PET 70%, median PFS 17 m; *N* = 39) [214–216]. Of note, there was no difference between BR-polatuzumab and BR alone possibly owing to a high discontinuation rate for peripheral neuropathy (grade 1–2 observed in 40%). The drug is also associated with approximately a 40% incidence of grade 1–2 diarrhea.

Mosunetuzumab was evaluated in FL (*n* = 69) with an ORR of 64% and CR of 44% [217]. The main concern with this agent is cytokine release syndrome (CRS), which is seen in approximately 25% of patients and is usually self-limited and responsive to standard of care management, including tocilizumab. Similar CD3-CD20 BiTEs are being investigated as monotherapy and in combination with other agents (e.g., lenalidomide, polatuzumab, and the anti-PD-L1 antibody atezolizumab) (NCT04246086, NCT03671018, NCT02500407). Magrolimab (anti-CD47 antibody) in combination with rituximab is associated with an ORR of 61% and CR 24% and a median time to response of 2 months with a favorable toxicity profile [218].

Finally, ibritumomab tiuxetan (Zevalin) is an anti-CD20 antibody linked to radioisotopes, which is FDA approved in FL and is not widely used due to logistical difficulties and concern for cytopenias and secondary myelodysplastic syndrome. While the overall PFS is only 9 months, PFS is nearly 4 years in the 35% of patients who achieve a CR [219]. The treatment is administered as a single infusion in the outpatient setting after two doses of rituximab. We reserve this treatment for patients who have exhausted other options.

### Indications for hematopoietic cell transplantation (HCT) and chimeric antigen receptor (CAR) T cells

The role of hematopoietic cell transplantation (HCT) in iNHL is controversial given the advent of novel agents and steady reductions over time in nonrelapse mortality (NRM) after HCT [220]. Consolidative autologous HCT (AHCT) has been most studied in patients with iNHL with POD24, where several reports have demonstrated a median PFS of 3–5 years, which is longer than the expected PFS with CIT alone and may potentially confer superior OS in high-risk patients who undergo AHCT within one year of initial treatment failure [221–223]. However, these data are limited by their retrospective nature, and AHCT has not been compared directly to novel treatments such as R^2^ [110, 224, 225]. With NRM rates less than 4% even in appropriately selected elderly patients, we offer AHCT consolidation to patients with refractory or early relapsed, high-risk FL who are in chemosensitive remission after salvage therapy, allowing us to reserve novel therapies for subsequent lines of treatment [221, 226].

The use and appropriate timing of allogeneic (allo)-HCT remains a complex treatment decision in patients with R/R iNHL, given concerns about high rates of NRM and graft-versus-host disease (GVHD) [227, 228]. In a large retrospective registry cohort of heavily pretreated patients with a history of POD24, allo-HCT was associated with impressive 5-year OS of 75% and 50% for matched related and unrelated donors, respectively, despite a median of three prior lines of therapy and 40% of patients transplanted with refractory disease [222]. However, there were considerable rates of NRM at 5 years (33% and 17% for matched related and unrelated donors, respectively), mainly secondary to GVHD [222]. It is plausible that with better patient selection and more modern HCT techniques, such as the use of reduced intensity (RIC) or non-myeloablative (NMA) conditioning, NRM would be considerably lower [220, 229, 230]. Haploidentical allo-HCT with posttransplantation cyclophosphamide (PT-Cy) has largely removed the barrier of donor availability with comparable efficacy and markedly lower rates of chronic GVHD compared to standard allo-HCT (12% vs. 49%, respectively) [231, 232]. We consider RIC or NMA allo-HCT, preferably on a clinical trial, for medically appropriate patients who relapse after AHCT and/or those with multiple relapses and short remission durations who demonstrate treatment sensitivity [233].

Decisions regarding HCT will become even more challenging with the forthcoming FDA approval of CD19-directed chimeric antigen receptor (CAR) T cells in iNHL. CAR T is associated with CR rates up to 88% with sustained remission in up to 89% of FL patients with an initial response, over median follow-up of 28.6 months [234–236]. Recently presented data from the phase 2 ZUMA-5 trial which evaluated commercially available axicabtagene ciloleucel in patients with FL and MZL demonstrated ORR 94% (95% in FL, 86% in MZL), with CR rate of 79% [237]. While high-grade CRS and neurotoxicity remain concerns, occurring at rates of 13% and 28%, respectively, our growing experience utilizing appropriately-timed anti-cytokine blockade and corticosteroids should help mitigate severe toxicities [234, 237, 238]. Future studies will evaluate CAR T cells for other iNHL histologies [239].

#### Conclusion

The treatment of iNHLs is one of the most fascinating and rapidly developing areas of oncology, in which clinicians need to balance potential toxicities with the benefits of treatment. An intimate familiarity with disease course and an understanding of molecular pathways are required to tailor therapy for each patient. The ideal treatment plan is one that will remain effective for many years while preserving the patients’ quality of life and longevity.

## Data Availability

Not applicable.

## Abbreviations

ADC: Antibody–drug conjugate
AHCT: Autologous hematopoietic cell transplantation
allo-HCT: Allogeneic hematopoietic cell transplantation
β2M: Beta-2-microglobulin
BALT: Bronchial-associated lymphoid tissue
BiTE: Bispecific T cell engagers
BM: Bone marrow
BNLI: British National Lymphoma Investigation
BR: Bendamustine–rituximab
CAR-T: Chimeric antigen receptor T cell
CIT: Chemoimmunotherapy
CNS: Central nervous system
CR: Complete response
ctDNA: Circulating tumor DNA
FFP: Freedom from progression
FL: Follicular lymphoma
FLIPI: Follicular Lymphoma International Prognostic Index
G: Obinutuzumab
GELF: Groupe d’Etude des Lymphomes Folliculaires
GI: Gastrointestinal
GVHD: Graft-versus-host-disease
HCL: Hairy cell leukemia
HCT: Hematopoietic cell transplantation
HCV: Hepatitis C virus
HTS: High-throughput sequencing
IFRT: Involved-field radiotherapy
ISRT: Involved-site radiotherapy
iNHL: Indolent non-Hodgkin lymphoma
LAD: Lymphadenopathy
MALT: Mucosa-associated lymphoid tissue
MCL: Mantle cell lymphoma
MRD: Minimal residual disease
MZL: Marginal zone lymphoma
NLPHL: Nodular lymphocyte-predominant Hodgkin lymphoma
ORR: Overall response rate
OS: Overall survival
PCFCL: Primary cutaneous follicle center lymphoma
PCMZL: Primary cutaneous marginal zone lymphoma
PET: Positron emission tomography
PI3Ki: PI3K-inhibitor
PJP: Pneumocystis jiroveci pneumonia
POD: Progression of disease
POD12/24: Progression of disease in 12/24 months
PR: Partial response
QoL: Quality of life
R: Rituximab
R^2^: Rituximab, lenalidomide
R/R: Relapsed/refractory
R-CHOP: Rituximab, cyclophosphamide, doxorubicin, vincristine, prednisone
R-CVP: Rituximab, cyclophosphamide, vincristine, prednisone
RM: Rituximab maintenance
RT: Radiotherapy
SMZL: Splenic marginal zone lymphoma
SUV: Standard uptake value
TTC: Time to first chemotherapy
TTF: Time to treatment failure
WBRT: Whole brain radiotherapy
WM: Waldenstrom macroglobulinemia
WW: Watchful waiting
